# Learners’ Perspective towards E-Exams during COVID-19 Outbreak: Evidence from Higher Educational Institutions of India and Saudi Arabia

**DOI:** 10.3390/ijerph18126534

**Published:** 2021-06-17

**Authors:** Mohammed Arshad Khan, Vivek Vivek, Maysoon Khojah, Mohammed Kamalun Nabi, Mohinder Paul, Syed Mohd. Minhaj

**Affiliations:** 1 Department of Accountancy, College of Administrative and Financial Sciences, Saudi Electronic University, Riyadh 11673, Saudi Arabia; m.khoja@seu.edu.sa; 2 Department of Commerce and Business Studies, Jamia Millia Islamia, New Delhi 110025, India; vs22.vivek@gmail.com (V.V.); mnabi@jmi.ac.in (M.K.N.); syedminhaj125@yahoo.com (S.M.M.); 3 Department of Commerce, Ramanujan College, University of Delhi, New Delhi 110019, India; paulmhndr@gmail.com

**Keywords:** online examinations, learners’ perception, higher education institutions, formative assessment, summative assessment, COVID-19

## Abstract

Online examinations, commonly referred to as e-exams (electronic examinations), underwent a considerable progression, getting adapted ubiquitously among higher education institutions worldwide. Their preferment was rapid due to the emergence of the COVID-19 pandemic. The process of conducting exams online is being opted as the appropriate way of assessment, ensuring the students’ safety and well-being. According to Warts et al., this form of examination has been pretty effective in the past when blended with the conventional assessment. However, at present, implemented as the singular way of assessment, e-exams have shown a more significant promise in being beneficial to the learners. As a matter of fact, a comprehensive analysis on understanding the learners’ perception towards the e-exams was not done earlier, particularly in the developing nations. Thus, it was pertinent to examine the pre-requisites of e-exams to promote it as a useful tool for the smooth conduct of exams in the aforesaid nations. Against such a backdrop, this study was conducted during January to March 2021 on 207 students enrolled in four universities, three situated in the National Capital Territory (NCT) of Delhi, India: Delhi University (DU), Jamia Millia Islamia (JMI), and Jawaharlal Nehru University (JNU), and one situated in Saudi Arabia, namely Saudi Electronic University (SEU). A quantitative approach was employed for the study, with the responses recorded via web questionnaires. Confirmatory -factor analysis (CFA) was applied in the study to examine whether the process of conducting online examinations is being chosen as the appropriate form of assessment, ensuring the safety and well-being of students through AMOS (version 24) software. For determining the reliability of the two latent constructs, namely “Perceptions of students towards E-exams (PSE)” and “Pre-requisites of E-exams (POE),” Cronbach’s alpha was used through SPSS (version 25) software in the study, and the results reveal that the strong internal consistency exists between all the measured variables. In addition, the mean and standard deviation were used by the researchers to find out the pre-requisites of the online examination system. The participants expressed their insights on the relative benefits of online examination. Their perception was based on pedagogy, validity and reliability, affective factors, practicality, and security. From their insights, it was concluded that online examination is more advantageous than conventional paper-based exams. The outcome also applies to the authenticity of grading and the overall efficiency concerning the time, effort, and expenditure on conducting the examination. Contrarily, the participating students also recognized numerous hurdles in implementing e-exams concerning security, validity, and impartiality. The conclusion further revealed that online examination is especially relevant for formative assessment of learning instead of summative assessment, provided authenticity, security, and flexibility are used as fundamental tenants in the proper implementation of e-exams. The outcome of the present study will facilitate higher education institutions and policymakers in taking the electronic examination system to the next level.

## 1. Introduction

In today’s era of digitalization, the term information and communication technology (ICT) has expanded to encompass many aspects of computing technology and is more recognizable than ever before. The increasing use of ICTs in academic institutions is attributed to several factors. For instance, employing ICTs in education shall improve the quality of education. It shall facilitate the absorption and acquisition of knowledge and improve the effectiveness of implementing educational policies [1]. E-exam refers to a computerized exam through which the student uses a computer for taking his/her exam. It serves as an effective, unbiased, and exciting method for assessing students’ academic level. Using e-exams for the assessment of students at the university level shall increase the objectivity of the assessment process [2,3].

Now there arises a question that often confuses most of us, i.e., what is the difference between computer-based exam and online exam? The term computer-based exam (or computer-based test) encompasses online exam. Online exams (also known as e-exams) may be considered as a sub-class of computer-based exam, wherein the exam is conducted online. Online is used in the sense that the computer being used to take the test is connected to the intranet or the Internet, in a client–server environment. The computer being used for taking the test can be considered a client computer. The server computer is the one that is sitting somewhere on the Internet (or the intranet) and delivering the exam. Usually, in an online exam, there is some form of communication between the server and the client (not necessarily continuous). The exam results are uploaded to the server computer after the exam. The role of the client comes to an end as soon as he/she completes the exam and the results are uploaded. In technical terms, the session ends after the exam is completed.

As far as computer-based exam is concerned, it is a generic term used for any kind of exam or test given on a computer. The computer may or may not be online. In other words, the computer may or may not be connected to a server on the Internet (or intranet). After a computer-based exam (CBE) or computer-based test (CBT), the results may be stored locally on the computer’s hard disk or transported online to the web server. Usually, standalone testing is referred to as CBT. Competitive exams are often referred to as online exams because they are delivered in real time simultaneously over the network to several students and the exams are conducted in a client–server environment. A CBT software exam engine may be viewed as an example of CBT. Here a candidate downloads the CBT software from the Internet and installs it on his/her own computer and appears in the exam. Here no server connection is required for appearing in the test/exam.

E-exam was introduced in the 1970s. It is used as an effective method for assessing one’s level of knowledge, academic performance, and problem-solving skills. It is employed as a research tool by researchers to collect data or by trainers and educators to assess students. It enables teachers to save their efforts while assessing students. E-exam has been increasingly used by higher education institutions worldwide because it provides relatively more accurate results by employing stimuli that may be in an audio, text, graphic, or kinesthetic form [4].

E-exams facilitate the simplification of the conventional, paper-based exams. They are particularly beneficial when the class sizes are considerable, enabling ease in devising and renditioning the process. It includes marking and reporting the examination, later on storing, and performing statistical analysis [5,6]. Besides, they constitute an entirely computerized process, which helps ameliorate the assessment validity, which further comprises the examinee’s performance, warranting a greater degree of skill and comprehension, for it employs enhanced question forms consolidating interactivity and multimedia. E-exams, furthermore, amend the authenticity of grading and the briskness of the exam results, promoting more extensive analysis and providing prompt feedback.

Compared to the conventional exam format, online examination substantially amends the efficacy of various data management responsibilities, comprising marking and moderation of the data, followed by secure storing, which overall reduces the workload of the teachers while also lessening the burden of invigilating large student cohorts [7]. Though e-exams are opted, by and large, learners’ perspectives on their application remain unplumbed. Accordingly, the primary objective of the present study is to evaluate the perception of learners having exposure to e-exam towards online examination methods of higher education institutions. The findings of this study will aid the universities in identifying the essential aspects of devising e-exams properly for the learners’ optimal benefit.

## 2. Statement of the Problem

Assessment is a vital part of the learning process. It is carried out for obtaining information about the learning outcomes of each student. The application of reliable assessment methods shall help to amend the effectiveness of the educational practices, and enable the students to distinguish whether they have met the required academic goals or not [2]. Due to the significance of online assessment during this phase of the ongoing pandemic, e-exams are employed at present to assess students’ performance. Many students must be assessed in various courses at universities within a limited amount of time through mid-exams and final exams. However, this will require use of a rapid assessment method that will provide scores instantly. Therefore, universities prefer to assess students through e-exams [8]. E-exams are being adopted internationally due to this ongoing pandemic. Therefore, this study investigates the effectiveness of the online examination system from the viewpoint of students enrolled in higher education institutions of India and Saudi Arabia.

## 3. Review of Literature

The earlier studies on understanding learners’ perceptions towards online examination are resourceful. They render a wide array of doubts shown by the students. However, the implication of such research is arguably limited, provided they were conducted during the pre-pandemic era. Students were mainly asked about their earlier experiences of assessment methods such as online practice tests and quizzes and their responses to various e-learning platforms (Google Classroom, Blackboard, Moodle, etc.). An earlier study was undertaken within a single discipline [9] to investigate the students’ perception towards online examinations at Ash-Shobak University College in Jordan. For this study, questionnaires comprising 26 items were distributed to 112 students. Of them, 108 questionnaires were collected and deemed to be adequate for the research. The outcomes were nifty, reflecting no disparity among the students’ perspectives concerning GPA or gender. The findings of the study revealed a positive outlook of students towards the online examination. Moreover, their take towards the authenticity and dependability of online exams was approving, stating that the electronic process is competent in evaluating what they intend to evaluate. The study also concluded that the online examination system and the respective regulations were transparent. However, in contradiction, it was discovered that online examinations create higher anxiety among students, and cheating becomes relatively easier. The research also concluded that the duration of online exams is improper, and the process does not help ameliorate the students’ performance.

Alsadoon [2] investigated students’ perception towards e-assessment at Saudi Electronic University in Saudi Arabia. A web-based survey comprising 15 items was conducted on 80 students registered in the aforesaid university. The survey was conducted during the academic year 2015–2016. This research deduced that students have a positive view towards e-assessment. The research, additionally, discovered that e-assessment amends the standard of learning and assessment methods while serving as an impartial system. It furthermore lessens the burden associated with exams, enhancing students’ technical abilities, and hinders cheating. Hence, students opt for being assessed via e-assessment instead of the conventional paper-based assessment.

Jamiludin [10] investigated high school students’ perception of national exams conducted through traditional paper-based exams and the modernized computer-based examination in Kendari, Indonesia. Interviews were held, and questionnaires consisting of 20 questions were provided to 34 students. The research deduced that the general preference of students was to take the traditional form of examination. The primary reason for their choice was the easier comprehension of the paper-based examination. The interviews, furthermore, concluded that the computer-based examination is beneficial in providing the students with a valuable experience of being accustomed to the technology. The process was also deemed time-efficient compared to the paper-based method and advantageous in hindering cheating. On the contrary, the study concluded that a computer-based examination negatively affects the students’ health because of prolonged screen viewing. Students also complained of their concentration taking a blow with the frequently fluctuating performance of the computer.

Alruwais et al. [8] examined the students’ perception towards computer-based exams. A total of 500 questionnaires were provided to students, selected from the Ladoke Akintola University of Technology in Nigeria, of which 400 questionnaires were collected and deemed valid for research. The analysis discerned that students have a favorable outlook towards the modern-day exam method, for it provides them with the option of editing their answers efficiently, serving as a secure assessment system. The study also revealed that students do not encounter any difficulty in logging in or out of the online exam application.

E-learning can take greater advantage of the various assessment systems, provided there is a lack of face-to-face communication between the teachers and the students, which otherwise can have valuable exchanges. Besides, for promoting rich e-learning participation, students’ progress can be measured to pass on relevant feedback and appropriate performance grading. The e-exams comprising multiple-choice questions (MCQs), true/false, or related questions are conducive in obtaining the essential information about learning in any assigned course. Reliable assessment practices such as online discussions, presentations, and assignments are helpful for a more extensive assessment of the students’ performance [11].

In the prevailing time, students and teachers have witnessed numerous applications of e-learning courses, and likewise, e-exams, resulting from the progression in ICT [12]. Learners have considerably enrolled in online courses, which seems to be appropriate given the benefits of cost-effectiveness, data storage, exam security, and a quicker result time. The primary benefit of saving time and reducing paper use adds up to it, along with the superior, automated record-keeping option for students, teachers, and institutions collectively [13].

Böhmer et al. [14] examined the perception of part-time engineering students towards the process of online examination. Their study revealed that the said students were largely content with the exam system. The reason behind their satisfaction includes the ease of participation in an online exam and quickly obtaining the result. In contemplating the learners’ presumptions on e-exams, Hillier [15] inspected undergraduate students. The findings of the research revealed that the majority of the students have a positive perception towards e-exams. The students, however, raised doubts, stating that the online exam method will appeal more to technology majors than the others. The research deduced that students believed that those belonging to the computer domain would enjoy the ease of adapting to the new system for having a substantial typing experience. Some of the students also expressed concerns related to possible technical failures and the likelihood of more cheating. A study conducted in the United Arab Emirates (UAE) by Elmehdi and Ibrahem [16] revealed a largely assertive outlook of students towards e-exams. Expedited logistics and advanced e-learning were the primary reasons for their positive response. Besides, the study revealed no disparity in their viewpoints concerning age and gender.

Bawarith et al. [17] employed an online examination management method to identify and hinder cheating. They used a fingerprint reader authenticator and an Eye Tribe tracker during the e-exam sessions to accomplish their objective. In one more scenario, Kolhar et al. [18] put forward an online lab examination management system (OLEMS) to hinder misconduct and safeguard the practice of lab examinations.

D’Souza and Siegfeldt [19] devised a compelling conceptual framework for identifying cheating during in-home or lab examinations. Cluskey et al. [20] put forward e-exam control procedures to ensure the safety and reliability of e-exams. The first of their procedures recommended that the examination be conducted simultaneously for everyone, with the access provided only through a particular web browser to ensure that the students are locked into the exam page and can refrain from exiting or returning, cutting or pasting data. Backman [21] further advised employing software to prevent Internet access and incorporating a question bank, where different students are given different questions. His study also suggested asking the students all the more challenging questions with limited response time to ensure that they do not think of cheating.

Thus, the literature review, by and large, revealed that learners had a positive outlook towards e-exams. However, the disparities as per the demographic variables were less evident. Besides, the literature review demonstrated harmony amongst the concerns expressed by learners belonging to both developed and developing nations. Often, the expressed concerns included the likelihood of technical glitches, increased cheating, inappropriate examination time, and a dearth in the questions’ quality.

## 4. Research Gap

The literature review revealed that most of the research studies are undertaken to showcase the learners’ perception towards the online examination. However, empirical studies to examine the perception of students towards e-exams conducted by the higher educational institutions situated in India and Saudi Arabia during the ongoing pandemic are few and far between. As a matter of fact, even though many studies on online learning and exams have been conducted by numerous researchers in the past, there is hardly any study that has been undertaken to cognize the essential considerations needed for the successful implementation of online exams in higher educational institutions of India and Saudi Arabia.

## 5. Objectives of the Study

The goals fundamentally sought after in this research are:

1. To investigate learners’ perception concerning the advantages of the online examination system towards the methods and practices of teaching, their authenticity and reliability, their practicality, the affective factors, and the security;
2. To identify all the necessary considerations required for the successful implementation of the online examination system in higher education institutions.

## 6. Research Methodology

This research study is descriptive-cum-cross-sectional in nature. For precise analysis, both the primary and the secondary data have been used. The purposive sampling (also known as judgmental sampling) method is a form of non-random sampling in which researchers rely on their own judgment when choosing members of the population to participate in their surveys. This sampling method requires researchers to have prior knowledge about the purpose of their studies so that they can choose and approach eligible participants for surveys. The purpose of the study was to assess the students’ perceptions of e-exams, and for this purpose, the researchers chose the students as per their own judgment of those institutions where the online examinations are being conducted by the universities in India and Saudi Arabia during the ongoing pandemic. Since the researchers have more prior information about the selected university students’ level of knowledge and understanding towards e-exams and how they perceive e-exams, it ensures the desired number of sample students that the researchers are going to select. Therefore, the judgmental sampling technique was applied by the researchers to obtain information from 207 students registered in the various universities of Delhi, India, and Saudi Arabia. These institutions were Delhi University (DU), Jamia Millia Islamia (JMI), and Jawaharlal Nehru University (JNU) from the NCT of Delhi, India, and Saudi Electronic University (SEU) from Saudi Arabia. The survey respondents are students enrolled in those universities conducting online exams during this phase of COVID-19 pandemic. The perspective of the learners towards the e-assessment questionnaire [22] was adopted for providing a framework for devising an online questionnaire comprising two principal sections: Section 1 and Section 2. The first section required filling up the general information. The second section, however, required students to answer several statements about their views on e-exams. All the statements were set forth positively, classified into six sets, which comprised the method and practices of teaching (also known as pedagogy), authenticity and reliability, practicality, affective factors, and security. Moreover, the section also comprised statements asking the students to choose the critical considerations required to implement e-exams successfully. Three experts in e-learning evaluated the content efficacy of the questionnaire and provided their opinions on the specified items. The experts, furthermore, recommended that specific revisions be made. The said revisions were minor in nature, and necessary rectifications were made accordingly. Besides, the authenticity of the constructs was analyzed using Cronbach’s alpha in the degree to which the items in the questionnaire were associated with one another. The alpha value of each construct used in the study is greater than 0.70, which shows that all the statements are significantly correlated with each other [23]. The survey was carried out from January to March 2021. For investigating whether the manifest variables clearly explain their corresponding construct, the CFA technique was conducted in the study with the help of AMOS (version 24) software. The researchers employed the appropriate statistical tools and techniques through SPSS (version 25) software to accomplish the primary objectives of the study.

## 7. Findings and Discussion

The web-based questionnaire shared by Google Forms was randomly provided to the students of various universities via multiple social networking sites. A significant number of students took part in this online survey. The web-based questionnaire was devised carefully, keeping in mind the variables under research. The questionnaire had closed-ended questions. A summated rating scale was used to garner the data from respondents. It was done based on a five-point basis, deviating from “strongly disagree (1)” to “strongly agree (5).” From the total collected responses of 234 students, 207 responses were found valid and considered for further data analysis. By using SPSS (version 25) software, the collected data were analyzed quantitatively. This section exhibits the results and findings of the research. It constitutes the demographic profile of the sample respondents, along with the device(s) that students utilize for giving the examination, their views towards the process, the needful considerations for the successful implementation of the online examination system, and the various problems and challenges encountered by the students/learners while appearing in e-exams.

### 7.1. Background Information of the Respondents

The general details of the students who filled the questionnaire are shown in this section. Table 1 displays the responses to the questions relating to various demographic variables chosen for the study. The information showcased here is gathered from the primary data.

The aforesaid table presents the demographic information on the participants classified on the basis of their gender, age group, course of study, level of study, and the institution where they were studying. It revealed that most of the sample respondents (61.4%) were females, whereas 38.6% were males. The above data also indicate that most of the students (49.8%) belonged to the age group of up to 20 years, 19.3% were aged between 21 and 25 years, and 16.4% fell within the age bracket of 26–30 years. At the same time, only 14.5% of the respondents were found to be above 30 years of age. For this majority age group of up to 20 years, the research findings are likely to reflect their youthful views.

Respondents are reasonably distributed based on their course of study. Table 1 indicates, out of a total of 207 respondents, 42.5% of the respondents were business students in general and accounting and marketing in particular, and there is a fair representation from the other courses of study as well—Art and Design 9.7%, IT 28%, and Engineering 19.8%. Thus, it can be concluded that there is a fair distribution of views from the various groupings of students in this study.

Furthermore, the collected dataset of sample respondents is justifiably distributed based on their academic year of study. A preponderance of the students (35.8%) was in the third year, followed by the first-year students (32.4%) and second-year students (18.8%), and the remaining 13% of the students were in the fourth year, respectively. Consequently, the present research comprises diverse groups of learners for attaining a good assortment of perceptions.

Besides, most of the students belonged to Jamia Millia Islamia University (33.3%), followed by Delhi University (31.9%), Saudi Electronic University (21.8%), and Jawaharlal Nehru University (13%), in that order. Thus, it signifies that a significant chunk of the sample respondents (78.2%) belongs to Indian universities and only 21.8% of the respondents belong to Saudi Electronic University of Saudi Arabia.

### 7.2. Reliability of the Latent Constructs

According to Hair et al. [24], “Cronbach’s alpha is the standard measure of internal correspondence between items in a scale, facilitating its widespread use with Likert scale-based questions used in the survey. The fundamental objective of reliability testing was to examine the attributes of the scales of measurement and the items for getting the overall index of internal consistency of the scales.” The outcomes of this test are provided in the (Table 2).

The above table showcases the reliability analysis of the latent constructs used in the study. All the measures depict the “high internal reliabilities” when the value of Cronbach’s alpha ranges between 0.70 and 0.90 since it surpasses the threshold limit of 0.70 [25,26]. It further affirms that the coefficient alpha of each latent construct is more than 0.90, indicating that there is strong internal consistency between the items on a scale chosen for the study.

### 7.3. Perception of Students towards the Online Examination System

**Figure 1:**
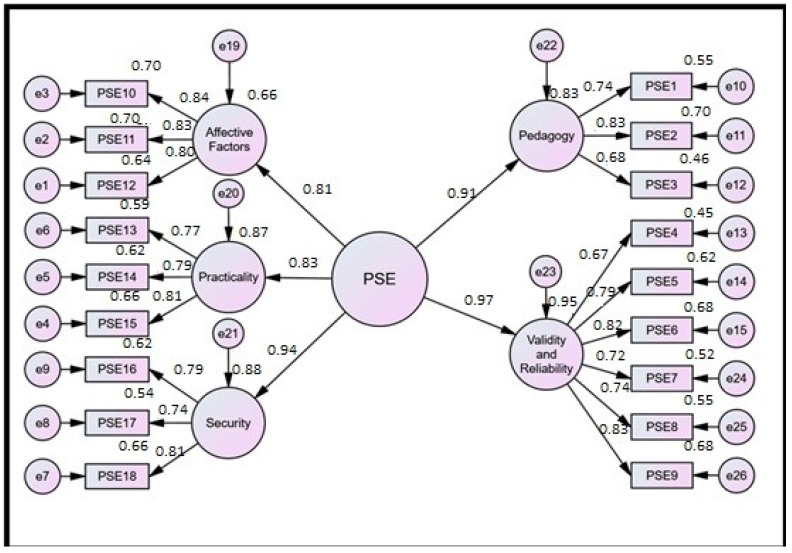
Perception of Students towards the Online Examination System.

To examine whether all the manifest variables clearly explain their respective latent construct, the researcher applied the confirmatory factor analysis (CFA) in the study via AMOS (version 24) software. According to this research study, in order to analyze the perspective of learners towards online exams, the major latent construct, namely “students’ perceptions of e-exams (PSE),” is categorized into five sub-constructs, and further, each of these sub-constructs is measured by various statements chosen by the researcher to collect responses from the participants. It is shown in the Figure 1.

The aforesaid model showcases the perception of students towards e-exams, the principal latent variable, measured by its five sub-constructs: pedagogy, validity and reliability, affective factors, practicality, and security. Pedagogy, the first sub-construct, is measured via three statements (PSE1, PSE2, and PSE3) expressed by rectangles, for they are observed variables. Validity and reliability, the second sub-construct, is measured via six items coded as PSE4, PSE5, PSE6, PSE7, PSE8, and PSE9. Affective factors, the third sub-construct, is examined by three items coded as PSE10, PSE11, and PSE12. Practicality, the fourth sub-construct, measured via three statements coded as PSE13, PSE14, and PSE15. Security, the fifth and final sub-construct, is analyzed via three items coded as PSE16, PSE17, and PSE18.

The small “e” represents error terms signifying the proportion of unexplained variation. The standardized regression coefficient for a particular item is denoted near the arrow leading to the respective item, while the value above each response item purports the squared multiple correlations (*R*^2^) of the manifest/measured variables. Table 3, Table 4 and Table 5 represent the analysis summary of the aforesaid model provided by Analysis of Moment Structure (v-24).

The above table depicts the chi-square (*χ*^2^) value, i.e., 0.157, which is greater than 5 percent, and the CMIN/DF value, i.e., 2.403, which is less than the recommended limit of 3. These values show that the garnered sample dataset is appropriate for the model fit. It furthermore produced four goodness-of-fit indices, i.e., GFI = 0.910, AGFI = 0.813, CFI = 0.927, and NFI = 0.913. These values surpass their acceptable limits, explaining that the model is a well-fitted model. The two badness-of-fit indices, i.e., RMSEA = 0.042 and SRMR = 0.046, are both less than the recommended limit, which shows that the collected sample dataset fits the model properly. Thus, it affirms that the aforesaid measurement model is a well-fitted model.

The above analysis shows that all the measured variables are significantly related to their corresponding constructs since their *p*-values are less than the recommended limit of 5% alpha level. Furthermore, the standardized regression weight (β) of each path is more than 0.40, which affirms that the convergent validity of the CFA measurement model discussed earlier is achieved and also illustrates that each manifest variable is highly correlated with its respective latent construct [27].

Table 5 shows that the composite reliability of each variable is greater than the threshold limit of 0.70, indicating that “strong internal consistency” exists between the items on a scale. Alternatively, the “average variance extracted” of each latent construct surpasses the recommended limit of 0.50. It asserts a “strong convergent validity” of the measurement model, as discussed above. This segment of the questionnaire was targeted for acquiring information about the students’ viewpoints towards e-exam methods exercised by the educational institutions in India and Saudi Arabia amid the COVID-19 pandemic. The principal headings were Pedagogy, Validity and Reliability, Affective Factors, Practicality, and Security. The respondents were requested to register their answers on a five-point summated scale varying from “strongly disagree (1)” to “strongly agree (5).” The respondents recorded mixed experiences of e-exams. Out of the 18 items of the questionnaire, 15 recorded positive mean responses, two recorded negative responses, and one recorded a neutral response (Table 6). The said rating is categorized into three categories: firstly, the mean score of 3 shows the neutral response of students; secondly, if the mean score is more than 3, it depicts the positive perception of students towards e-exams; and lastly, if the mean value is less than 3, it portrays the negative mindset of students regarding the online examination system [22]. The following table depicts the results:

Table 6 indicates that the overall mean value is 3.74, and the value of the overall standard deviation is 0.881. It reveals that university students’ perception towards online exams is positive as the total mean value is greater than 3 and the total standard deviation is less than 1, which is considered to be stable. In fact, it is consistent with the results of Tella and Bashorun [28] and Da’asin [9]. This may be associated with the fact that respondents have excellent computer skills, enabling them to use the e-exam system with ease.

**Pedagogy:** It was discovered that students agree that immediate feedback in online exams helps them understand the subject better. Moreover, the cutting-edge technology used in e-exams facilitates the students in adapting the online learning approach rather than the traditional approach of pen–paper-based ones. The mean values and the standard deviation of all the three statements are greater than 3 and less than 1, respectively. This is consistent with the results of Chin et al. [29]. This may also be associated with the fact that students today prefer using technology as compared to conventional methods. Using a computer for assessment shall make students feel that they are keeping up with the technology.

**Validity and Reliability:** It is one of the most critical elements of online assessment. Table 6 shows that the mean score of the statement coded as PSE4 is less than 3, which reveals that the students think online exams are not valid since they are inappropriate for various fields of studies and subjects. It was found that e-exam is an effective method for assessing one’s level of knowledge. That is because the mean score of the statement coded as PSE5 is 3.83. This is consistent with what is suggested by Daramola [4]. That is because an examinee who has studied well for an exam can answer any question regardless of its form.

Furthermore, Table 6 shows how the e-exams promote more reliable assessment compared to the paper-based method of examination via integrating multimedia and simulations, provided the mean value concerning this statement (PSE6) is greater than three. A related outcome is registered by Kuikka et al. [30]. Their conclusion revealed that modern-day technology accommodates for examinees to be accustomed to video, audio, or simulations before responding to various kinds of questions relating to multimedia, hence making e-exams all the more appealing than paper-based methods. Students like exploiting technology for transforming the assessment practices while ensuring the assessment is authentic. They do so via strategies, such as problem-based approaches, portfolios of evidence, simulation, and the integration of online and face-to-face assessment [7].

Moreover, when the learners were enquired if they believe that the employment of e-exams will amend the exactitude of the results, most of them agreed to the statement, emphasizing that an automated marking system is far more precise than the traditional system. They further opined that the online examination system is impartial and has “no bias in grading.” The said finding is in association with the analysis by Baleni [31], who discovered that transparency in marking and prompt deliveries of grades bestow students with more confidence in comparison with the time-taking paper-based method.

As far as whether the online examination is more valid and reliable than paper-based exams is concerned, students were neutral, which means they neither agreed nor disagreed with this statement coded as PSE8 since the mean value is exactly 3.00, which is considered to be an indifferent opinion of the participants included in the online survey. However, the online exam system offers speedy and accurate solutions within the desired time limit compared to the pen–paper test because the mean score of this statement (PSE9) is 4.41, and the value of standard deviation is less than 1.

**Affective Factors:** Another critical area of concern was the affective aspects of e-exams. It was found that students appearing in an e-exam feel less stressed than those appearing in a paper-based exam. That is because the mean of the statement coded as PSE10 is 3.30. This is consistent with the results of Da’asin [9], attributed to the way of presenting questions in the e-exam system. For instance, in an e-exam, each question is usually presented on a separate page. Thus, when the student does not see all the questions together, he/she shall feel less stressed. It was observed that because of e-exam, students feel comfortable to concentrate in the exam. That is because the mean of the statement coded as PSE11 is 3.18. This is consistent with the results of Chin et al. [29]. The e-exam may include different colors, multimedia, and simulation models that shall attract students’ attention and keep them focused. Even the students feel more comfortable appearing in an online exam than a pen–paper-based one as the mean score of this statement is 3.43, which is more than 3, considered neutral on a five-point summated scale.

**Practicality:** The research outcomes reveal that most of the students admitted to e-exams are comparatively more efficacious than pen–paper format, concerning time, efforts, and the cost. This outcome is achieved as the mean value is more than three, which is deemed as neutral on a five-point Likert scale. The wholly computerized modern-day system enables elimination or simplification in the printing, grading, result analysis, invigilation, and the staff workload, required for large class size. The said outcome comes in association with numerous other research studies [22,31]. However, it is necessary to first migrate from the traditional form of learning to the new-found digital approach to pedagogy and learning, which is time-consuming and expensive, particularly in the initial stage of implementation, to make appropriate utilization of the e-learning system for the reduction of staff workload [30].

One of the significant benefits of e-exams comes from the advanced formulation of a question bank, which serves as a ready reckoner from the examination’s perspective, provided the mean value is 4.12. However, it is necessary to be regular in renewing the question bank to minimize cheating, which can be done via memorizing the recurring questions. Moreover, the questions selected for e-exams must undergo quality assurance standards to make sure the devised questions align with the learning objectives of the course. For instance, concocting multiple-choice questions (MCQs) becomes significantly time-consuming for the management. It requires considerable technical and pedagogical expertise and assistance. When enquired about the convenience of online examination, most of the students acknowledged the same, indicating the superiority of e-exams over the traditional format. That is because e-exams can be attempted from any place and at any time, which is also applicable to online lectures, with smartphones often acting as the carriers; it is essential that formative testing be carried out regularly [32]. Another important circumstance is the notable progression of e-learning, also known as distance education. The institutional infrastructure in Palestine has undergone a significant change whereby universities are, to varying degrees, adopting e-learning and conducting e-exams [32]. As more and more universities adopt this new system, appropriate attention needs to be given to the proper implementation of e-exams. Besides, it is also required for the universities to improve their infrastructure and address security issues.

**Security:** It is a critical aspect of any examination. This section explicates that test materials and results of e-exams are more secure than those of traditional methods. Even in the online examination system, we can set up an automated timer for the whole exam or per question, which means that online exams are more secure than pen–paper-based ones. That is because the mean values of both the statements coded as PSE16 and PSE18 are 3.45 and 4.12, respectively.

Furthermore, the strongest negative response (mean = 1.67) asserted that the technology of e-exams is adequately competent in ensuring no cheating or plagiarism. Towards this, the respondents mostly disagreed. It is quite challenging to prevent cheating in an e-exam, provided technologies, such as smartphones, wireless networking, and Bluetooth devices, are readily available to everyone. It accommodates various means for the students to gain Internet access and search for the information needed during an online exam. Moreover, it is easier to pass on the gained information through wearable tech, which is not easy to prevent students from accessing. In scenarios when larger groups of students are taking the examination at different sets of times, one group of learners can easily pass on relevant information to the other, spoiling the reliability of such exams. Consequently, it is difficult to prevent students from cheating during e-exams, especially in the technologically advanced time we live in.

### 7.4. Pre-Requisites of an Online Examination System

Numerous considerations deemed efficacious concerning the rightful progression and application of e-exams criteria are also listed in the survey. Drawing on the five-point summated scale that varies from “strongly disagree (1)” to “strongly agree (5),” requests were made to the students to express their opinions on the necessities of e-exam methods exercised by the educational institutions in India and Saudi Arabia. The collective response of “agree” was consolidated by clubbing the two expressions “strongly agree” and “agree.” Identically, the method is applied to “strongly disagree” and “disagree,” constituting a collective response of “disagree,” whereas the “neutral” expression was left unaltered. Table 7 shows the results of this section.

Table 7 depicts the respondents’ outlook concerning the pre-requisites of e-exam practices adopted by the universities to ameliorate their examination procedure during this unprecedented phase of the COVID-19 pandemic. The above-described research explicates that the partaking learners considered that the presently exercised e-exam criteria adopted by the various universities during the COVID-19 pandemic are satisfactory. Furthermore, it can also be comprehended that the learners underline multiple beneficial features of online examination, most notably the authenticity in the grading process and the greater efficiency that comes in the time, effort, and cost. Hence, the survey results reveal that numerous potential amendments to the criteria of e-exams have been successfully identified amidst several educational institutions, more specifically among the Indian and Saudi Arabian universities, concerning the learners’ perception.

**Online Exam Design:** The results show that 43.4% of the respondents agreed that universities should maintain a question bank including different sets of validated questions to conduct their online exams efficiently, while only 14.6% of the respondents disagreed to the proposition. However, 42% of the respondents were neutral regarding this survey item, which means they were undecided regarding whether the universities should maintain a question bank.

Furthermore, 49.7% of the respondents opined developing different essential questioning techniques such as objective-type questions or MCQs, very short answer and short answer questions, long answer essay-type questions while conducting e-exams, and 35.3% were neutral. However, 15% of the respondents disagreed with asking different types of questions in the online exams. Besides, most of the respondents felt that online exams should be devised in such a way by the colleges and universities that will provide immediate significant feedback to the students against their responses.

**Online Exam Security:** The research findings outlined in Table 7 show that 61.3% of the respondents opined that the universities should keep their e-exams confidential, while 25.6% of the respondents were undecided, and the remaining 13.1% of the respondents were arguing with this statement coded as POE4. Furthermore, approximately 49% of the student respondents agreed that the online exams must be authentic and such kind of examination practice should not facilitate cheating. However, 16.4% and 11.5% of the respondents disagreed with the authentication and cheating aspect, respectively. Regarding neutral responses of the participants, 35.3% of the students were indifferent in respect of minimizing cheating consideration, and 39.6% were undecided regarding the authentication issue. Moreover, the universities must critically think about the security issue in the online examination system because, in this e-environment, numerous universities are adopting this innovative technique of conducting exams. It would be conducive to them for maintaining their academic integrity in the education world.

**Online Exam Purpose:** The researchers sought to determine the purpose for which the online exams should be conducted in the universities. In today’s era of e-learning, it is pertinent to examine the need for an online examination system because it would be conducive to education institutions for implementing e-exams. Based on the findings presented in Table 7, 53.1% of the respondents indicated that there should be an evaluation of student learning progress and achievement while using the platform of e-learning as it is an essential consideration for conducting the online exams effectively. However, 31.9% of the respondents were neutral, and 15% of the respondents disagreed to assessing students’ learning outcomes during the ongoing lesson, unit, or course.

Besides, the universities should also link the e-exams to intended learning outcomes (ILOs) as is necessary for a good program and unit planning and assessment of the students. Regarding ILOs, 48.3% of the respondents opined that the universities must include these learning outcomes in online exams. That is because intended learning outcomes (ILOs) define what the students have acquired and can do upon completing their course/studies. In addition to this, 37.7% of the respondents were neutral, and 14% disagreed to the statement coded as POE8.

It was also discovered that 54.1% of the respondents agreed with the statement, i.e., “linking analysis of results to quality assurance criteria” is an essential consideration for successful implementation of e-exams since quality assurance is a way of preventing mistakes and defects in the online examination system. By contrast, only 11.1% of the respondents denied that the implementation of inspection and structured testing to measure quality assurance is not essential to implement the e-exams efficiently and effectively, whereas 34.8% of the respondents preferred to remain neutral.

**Institutional Support:** The findings of the study also revealed that information and communication technology (ICT) is a business imperative these days. Technology has changed every sector, and the ageing education field is no exception. That is because 53.6% of the respondents believed that an online examination system must be aligned with the university’s long-term strategic plan. However, 36.7% of the respondents were neutral, and the rest, only 9.7% of the respondents, disagreed to the statement coded as POE10.

The findings also revealed that 58.9% of the respondents asserted that the universities should raise the requisite resources and make students aware about the essential guidelines required to attend the e-exams successfully. However, 26.6% of the respondents were undecided and the remaining 14.5% of the respondents disagreed with the initiatives taken by the universities for the convenience of students.

As far as the last statement (coded as POE12) is concerned, 59.4% of the respondents were of the opinion that the higher education institutions should provide proper guidance and support to the teachers and students since it is imperative for conducting the e-exams effectively. However, 27.5% of the respondents were neutral, and the remaining 13.1% of the respondents were not in favor of these considerations taken by the academic institutions for the successful implementation of online exams.

## 8. Conclusions

Online assessment is gaining more and more popularity during the ongoing COVID-19 pandemic. Its benefits are very encouraging for students, teachers, and universities as well. In this competitive era, every university is looking towards qualitative and cost-effective methods of examination. During this COVID-19 outbreak, it has become imperative for universities to adopt the online examination system. However, it is similar to a twin-edged sword, having both benefits and problems. The universities should carefully design their e-exam strategy to reap the benefits of the technology and the students’ needs concurrently.

Besides the growing implementation of online learning platforms in the academic institutions, this research also explored its viability, identifying various factors necessary for consideration before migrating to the online examination system, based on the perceptions of students enrolled in Indian and Saudi Arabian universities. Students/learners recognized various benefits of e-exams compared to the paper-based method, including critical factors of reliability in scoring and long-term effectiveness concerning time, effort, and cost. The research findings emphasized impartiality, authenticity, and security being the primary challenges meeting the successful implementation of e-exams. Exercising the automated assessment system warrants proper association of the academic and technical units. At first, devising a question bank for e-exams demands extra efforts. The questions must measure up to the proposed level of knowledge. The teachers need to have adequate training to organize online courses and examinations appropriately. The organizational units must promote a teaching–learning environment and provide the requisite structure for the system. Furthermore, e-learning and the online examination system work effectively through modern-day technologies, such as computers, network devices, and so forth.

Bugbee [33] advises the exam developers to explain e-exams and pen–paper exams as equivalent or render scaling information for comparison. The majority of the teachers and instructional advisors lack the expertise required in managing the online examination judiciously. The research findings reveal that computerized education, even with identical items, will not yield similar student learning standards. Teachers and educational institutions must employ the time, effort, and money needed to produce a positive outlook towards electronic assessment. The efficacy of e-exams can then, therefore, be attained by devising them to be authentic, reliable, secure, and compliant in promoting learning and ensuring alignment with intended learning outcomes (ILOs).

Hence, for a triumphant implementation of e-exams, higher education institutions must showcase support, including the formulation of adequate conditions required for conducting e-exams in the universities, attributing to the needfulness of training the students for using the online assessment system. Otherwise, they will experience anxiety while appearing in such examinations. To circumvent such a problem, the students must be well acquainted with the online assessment system and have a jolly atmosphere when appearing in the e-exams. It also expedites the administrative processes, rendering the required monetary assistance, amending the infrastructure, and strengthening the magnitude of the academic staff while equipping them with the appropriate guidance and the necessary technological and pedagogical support. The research findings further revealed that the e-exam method must be embedded in the university’s strategic planning for sustainable development.

## Figures and Tables

**Table 1:** Baseline Data of the Participants (*N* = 207).

Basis	Categories	*f*	*cf*	%
**Gender**	Male	80	80	38.6
	Female	127	207	61.4
**Age Group**	Up to 20 years	103	103	49.8
	21–25 years	40	143	19.3
	26–30 years	34	177	16.4
	Above 30 years	30	207	14.5
**Academic Courses**	IT (IT, Computer Science, Statistics, and Mathematics)	58	58	28.0
	Engineering (Agricultural, Mechanical, Civil, and Electrical)	41	99	19.8
	Business (Accounting, Secretarial, Supply Chain and Marketing)	88	187	42.5
	Art and Design (Fashion and Industrial Art)	20	207	9.7
**Level of Study**	1st year	67	67	32.4
	2nd year	39	106	18.8
	3rd year	74	180	35.8
	4th year	27	207	13.0
**Sources of Data**	Delhi University	66	66	31.9
	Jamia Millia Islamia	69	135	33.3
	Jawaharlal Nehru University	27	162	13.0
	Saudi Electronic University	45	207	21.8

**Table 2:** Reliability Analysis.

Construct	A	*f*
Students’ Perceptions of E-exams (PSE)	0.952	18
Pre-Requisites of E-exams (POE)	0.949	12

**Table 3:** Model Fit Analysis.

Name of Category	Required Fit Indices	Acceptable Limits	Values Obtained
Absolute Fit Indices	*χ* ^2^	*p*-value > 0.05	0.157
	RMSEA	<0.05	0.042
	SRMR	<0.09	0.046
	GFI	>0.90	0.910
Incremental Fit Indices	AGFI	>0.80	0.813
	CFI	>0.90	0.927
	TLI	>0.90	0.922
	NFI	>0.90	0.913
Parsimonious Fit Index	CMIN/DF	<3	2.403

**Table 4:** Analysis Summary of Scalar Estimates.

Path	β	*R* ^2^	CR	*p*-Value
PSE → Pedagogy	0.913	0.834	8.794	<0.001
PSE → Validity and Reliability	0.973	0.947	9.667	<0.001
PSE → Affective Factors	0.811	0.657	8.631	<0.001
PSE → Practicality	0.932	0.869	9.637	<0.001
PSE → Security	0.939	0.881	9.662	<0.001
Pedagogy → PSE1	0.742	0.550	12.274	<0.001
Pedagogy → PSE2	0.834	0.696	11.557	<0.001
Pedagogy → PSE3	0.681	0.464	9.431	<0.001
Validity and Reliability → PSE4	0.668	0.447	10.572	<0.001
Validity and Reliability → PSE5	0.786	0.618	10.137	<0.001
Validity and Reliability → PSE6	0.824	0.679	10.550	<0.001
Validity and Reliability → PSE7	0.719	0.517	9.374	<0.001
Validity and Reliability → PSE8	0.743	0.552	9.650	<0.001
Validity and Reliability → PSE9	0.827	0.684	10.583	<0.001
Affective Factors → PSE10	0.835	0.698	12.789	<0.001
Affective Factors → PSE11	0.834	0.695	12.769	<0.001
Affective Factors → PSE12	0.799	0.638	12.337	<0.001
Practicality → PSE13	0.766	0.587	12.036	<0.001
Practicality → PSE14	0.786	0.618	12.441	<0.001
Practicality → PSE15	0.815	0.664	12.298	<0.001
Security → PSE16	0.788	0.621	12.447	<0.001
Security → PSE17	0.735	0.540	11.383	<0.001
Security → PSE18	0.813	0.660	11.284	<0.001

β= Beta Coefficient, R^2^= Squared Multiple Correlations, and CR= Critical Ratio.

**Table 5:** Construct Validity Results.

Construct	Composite Reliability	Average Variance Extracted
Pedagogy	0.797	0.570
Validity and Reliability	0.893	0.583
Affective Factors	0.864	0.680
Practicality	0.833	0.623
Security	0.823	0.607

**Table 6:** Perception of Students towards Online Exams.

Code	Statements	Mean	Standard Deviation
**I. Pedagogy**			
**PSE1**	Immediate feedback in online exams helps students to get a deeper understanding of the subject.	4.35	0.872
**PSE2**	Using cutting-edge technology in online exams enables students to take a new learning approach, i.e., online learning.	4.43	0.724
**PSE3**	Online exams facilitate a more adaptive learning approach than pen–paper-based ones.	4.07	0.289
**II. Validity and Reliability**			
**PSE4**	Online exams are appropriate for any subject area.	2.89	1.261
**PSE5**	Online exams are felicitous to test the learners’ level of knowledge.	3.83	0.675
**PSE6**	Online exams facilitate more authentic assessment than traditional methods through integration of multimedia, simulations etc.	3.27	1.098
**PSE7**	Automated grading in the online examination system is more convenient and authentic than the standard grading method.	4.31	0.376
**PSE8**	Online exams are more valid and reliable than pen–paper-based exams.	3.00	1.201
**PSE9**	Online exam system offers speedy and accurate solutions within the desired time limit compared to pen–paper tests.	4.41	0.752
**III. Affective Factors**			
**PSE10**	Online examinations reduce stress and exam anxiety.	3.30	1.237
**PSE11**	Using online exams allows students to focus and concentrate more on the questions.	3.18	1.155
**PSE12**	Students feel more comfortable while appearing in an online exam than a pen–paper-based one.	3.43	1.031
**IV. Practicality**			
**PSE13**	Online exams are more efficient in terms of time, effort and money spent.	4.62	0.670
**PSE14**	Creating a question bank will act as a ready reckoner from an exam point of view.	4.12	0.940
**PSE15**	Online exams are more accessible than pen–paper-based exams.	3.29	1.142
**V. Security**			
**PSE16**	Test materials and results of online exams are more secure than traditional methods.	3.45	1.156
**PSE17**	The technology used in online exams is sufficiently effective in dealing with cheating and plagiarism.	1.67	1.237
**PSE18**	Setting up an automated timer for the whole exam or per question means that online exams are more secure than pen–paper-based ones.	4.12	0.382
	**TOTAL**	3.74	0.881

**Table 7:** Considerations for the Effective Implementation of E-exams.

Code	Statements	SD (1)	D (2)	Total (1 + 2)	N (3)	A (4)	SA (5)	Total (4 + 5)
**I. Online Exam Design**								
POE1	Maintaining a question bank including different sets of validated questions for adaptive testing.	6.3%	8.3%	**14.6%**	42.0%	32.9%	10.5%	**43.4%**
POE2	Developing the different essential questioning techniques while conducting online exams.	6.8%	8.2%	**15.0%**	35.3%	39.1%	10.6%	**49.7%**
POE3	Provide immediate meaningful feedback.	6.3%	7.7%	**14%**	24.6%	46.9%	14.5%	**61.4%**
**II. Online Exam Security**								
POE4	Maintaining confidentiality.	6.3%	6.8%	**13.1%**	25.6%	42.0%	19.3%	**61.3%**
POE5	Minimizing cheating.	7.2%	9.2%	**16.4%**	35.3%	28.5%	19.8%	**48.3%**
POE6	Authentication.	7.2%	4.3%	**11.5%**	39.6%	33.8%	15.1%	**48.9%**
**III. Online Exam Purpose**								
POE7	Evaluation of student learning progress and achievement during the ongoing lesson, unit, or course.	7.3%	7.7%	**15.0%**	31.9%	40.1%	13.0%	**53.1%**
POE8	Linking online exams to intended learning outcomes (ILOs).	8.2%	5.8%	**14.0%**	37.7%	35.3%	13.0%	**48.3%**
POE9	Linking analysis of results to quality assurance criteria.	6.8%	4.3%	**11.1%**	34.8%	44.0%	10.1%	**54.1%**
**IV. Institutional Support**								
POE10	Integrating the online exam within the strategic plan.	5.4%	4.3%	**9.7%**	36.7%	42.0%	11.6%	**53.6%**
POE11	Providing resources and facilitating procedures.	7.2%	7.3%	**14.5%**	26.6%	42.5%	16.4%	**58.9%**
POE12	Providing support for teachers and students.	6.3%	6.8%	**13.1%**	27.5%	40.1%	19.3%	**59.4%**

## Data Availability

This study supports the findings of the research study available online at https://dergipark.org.tr/tr/download/article-file/844448 (accessed on 24 December 2020).
